# Obstructive Sleep Apnea Resolution in Hypopnea-Predominant versus Apnea-Predominant Patients after Maxillomandibular Advancement

**DOI:** 10.3390/jcm12010311

**Published:** 2022-12-30

**Authors:** Jean-Pierre T. F. Ho, Ning Zhou, Jan de Lange

**Affiliations:** 1 Department of Oral and Maxillofacial Surgery, Amsterdam UMC, University of Amsterdam, Meibergdreef 9, 1105 AZ Amsterdam, The Netherlands; 2 Academic Centre for Dentistry Amsterdam (ACTA), University of Amsterdam and Vrije Universiteit Amsterdam, 1081 LA Amsterdam, The Netherlands; 3 Department of Oral and Maxillofacial Surgery, Northwest Clinics, 1815 JD Alkmaar, The Netherlands; 4 Department of Orofacial Pain and Dysfunction, Academic Centre for Dentistry Amsterdam (ACTA), University of Amsterdam and Vrije Universiteit Amsterdam, 1081 LA Amsterdam, The Netherlands

**Keywords:** obstructive sleep apnea, maxillomandibular advancement, surgical outcome, apnea, hypopnea

## Abstract

This retrospective cohort study aimed: (1) to analyze the influence of apnea-predominant versus hypopnea-predominant obstructive sleep apnea (OSA) on surgical outcome after maxillomandibular advancement (MMA); and (2) to evaluate whether MMA alters the presence of apnea-predominant to hypopnea-predominant OSA more than vice versa. In total 96 consecutive moderate to severe OSA patients, who underwent MMA between 2010 and 2021, were included. The baseline apnea–hypopnea index, apnea index, and oxygen desaturation index were significantly higher in apnea-predominant group, while the hypopnea index was significantly higher in hypopnea-predominant group (*p* < 0.001). No significant difference was found between apnea-predominant group and hypopnea-predominant group in the degree of advancement of A-point, B-point, and pogonion. Surgical success and cure were significantly higher in the hypopnea-predominant group compared to the apnea-predominant group, 57.4% versus 82.1% (*p* = 0.021) and 13.2% versus 55.5% (*p* = 0.012), respectively. Of the 68 (70.8%) apnea-predominant patients, 37 (54.4%) shifted to hypopnea-predominant after MMA. Of the 28 (29.2%) hypopnea-predominant patients, 7 (25%) shifted to apnea-predominant postoperatively. These findings suggest that preoperative hypopnea-predominant OSA patients might be more suitable candidates for MMA compared to preoperative apnea-predominant OSA patients. Additionally, MMA proved to alter the presence of apnea-predominant to hypopnea-predominant OSA to a larger extend than vice versa.

## 1. Introduction

Obstructive sleep apnea (OSA) is a sleep breathing disorder, where patients have recurrent episodes of partial or complete upper airway collapse and obstruction. This leads to periods of absent and/or reduced respirations during sleep, called apneas and hypopneas, respectively [1]. Both of these two events result in brief periods of arousal, which in turn leads to marked sleep fragmentation [2]. The apnea–hypopnea index (AHI)—calculated by the number of apneas and hypopneas per hour of sleep—has been criticized for decades, however to this day it is still used to diagnose, objectively measure and express the severity of OSA, and to evaluate desired treatment effect [3]. The American Academy of Sleep Medicine defines an apnea in adults as a decline of the peak signal excursion by ≥90% of pre-event baseline for ≥10 s and a hypopnea in adults as a decline of the peak signal excursions by ≥30% of pre-event baseline for ≥10 s in association with either ≥3% arterial oxygen desaturation or an arousal [4,5].

The economic burden of OSA is believed to be immense, with an estimated 1.4 billion adults between the age of 30 and 69 years suffering from OSA globally [6]. The estimated cost in the United States of OSA related motor vehicle collisions in the year 2000 was estimated to be 15.9 billion dollars [7]. Additionally, sleep disorders are believed to cost approximately 130 billion dollars annually in the United States [8]. In addition to these economic aspects, the health-related burden of OSA is also quite compelling, with OSA being associated with cardiovascular disease, metabolic disease, psychiatric disorders, and neurocognitive impairment [9,10,11]. For these reasons it is crucial that physicians are not only able to recognize OSA, but also are able to suggest and/or provide adequate treatment to these patients.

Continuous positive airway pressure (CPAP) is considered the first-line treatment for moderate to severe OSA, due to its high efficacy and noninvasive character [12,13,14]. A major disadvantage, however, is the fact that the adherence to CPAP has proven to be poor specifically in the long term [15,16]. For these patients—who cannot tolerate and/or are unable to adhere to CPAP—maxillomandibular advancement (MMA) might provide an alternative treatment option with success rates similar to that of CPAP [13,17,18]. However, no matter how efficient MMA has proven to be, there is still up to approximately 15% of MMA patients that are considered non-responders [18].

The identification of specific risk factors and/or predictors for treatment success are essential for physicians in order to adequately treat patients and to optimally utilized resources. Previous research has established that certain factors are associated with a higher MMA success rate, i.e., lower body mass index, younger age, smaller neck circumference, and greater maxillary advancement [19,20]. Additionally, cardiovascular disease, complete anteroposterior epiglottic collapse found during drug induced sleep endoscopy, and larger superior posterior airway space seen on lateral cephalograms all were found to be associated with lower success rates [21,22]. Specific findings on preoperative polysomnography—e.g., AHI, positional dependency, central and mixed apnea index—have been investigated in order to see whether they were predictive for MMA outcome [19,22,23,24]. However, what is not yet clear is the impact of hypopnea-predominance versus apnea-predominance in the AHI on outcome after MMA.

Therefore, the aim of this study was (1) to analyze whether hypopnea-predominant OSA was more likely to have better resolution of OSA when compared to apnea-predominant OSA after MMA; and (2) to evaluate whether MMA alters the presence of apnea-predominant OSA to hypopnea-predominant OSA after MMA and vice versa.

## 2. Materials and Methods

### 2.1. Ethical Considerations

This study was deemed not to be subject to the Medical Research Human Subjects Act by the Medical Ethics Committee of the Amsterdam University Medical Centers (UMC), location Academic Medical Center (AMC) (reference number W22_269 # 22.328). Therefore, a formal approval was waived. Patients were sent a letter to inform them that their medical records, polysomnography results and radiological images were going to be used for study purposes. They were given the option to object and opt out of inclusion in the study. This study was performed in accordance with the Declaration of Helsinki guidelines for human research, 1964, and amended in 2013 (64th WMA General Assembly, Fortaleza, Brazil). It was conducted at the Department of Oral and Maxillofacial Surgery of the Amsterdam UMC, location AMC, The Netherlands.

### 2.2. Study Participants

We performed a single-center retrospective study including a consecutive series of patients with OSA undergoing MMA surgery between 2010 and 2021 at the Department of Oral and Maxillofacial Surgery at the Amsterdam UMC, location AMC. Patients who met the inclusion criteria were eligible for this study. The inclusion criteria were: adults aged ≥ 18 years; (2) diagnosis of moderate to severe OSA (apnea–hypopnea index [AHI] ≥ 15 events/hour) as determined by a preoperative overnight polysomnography (PSG); (3) continuous positive airway pressure (CPAP) therapy failure or intolerance; (4) presence of a follow-up PSG at least 3 months postoperatively. The exclusion criteria were: (1) No consent to the use of the patient record data for research purposes; (2) absence of apnea index (AI) and/or hypopnea index (HI) in preoperative and/or postoperative PSG report; (3) patients who underwent other adjunctive procedures at the time of MMA (e.g., multi-piece Le Fort osteotomy, temporomandibular joint reconstruction); (4) previous history of Le Fort I osteotomy or bilateral sagittal split osteotomy (BSSO); and (5) cleft palate and/or craniofacial syndromic patients. The included medical records were reviewed, and data were collected. Preoperative (baseline) patient data included gender, age, and body mass index (BMI).

### 2.3. Maxillomandibular Advancement Surgery

All MMA procedures were completed using standardized surgical techniques by two dedicated surgeons, which included a Le Fort I osteotomy for the maxilla in combination with a Hunsuck–Dal Pont modification of the Obwegeser BSSO for the mandible. Both maxilla and mandible were advanced anteriorly and whenever possible counterclockwise rotated [25].

Prior to the availability of three-dimensional (3D) planning, patients were treated with a traditional two-dimensionally planned surgical procedure with manually manufactured intraoperative occlusal splints. After the availability of 3D planning, patients were virtually planned and computer-aided design/computer-aided manufacturing intraoperative occlusal splints were used [25,26].

Immediately postoperatively, all patients received extensive postoperative monitoring in either the intensive or medium care unit [25,27,28,29]. After being discharged from the intensive or medium care unit, the patients were transferred to a general post-surgery ward for further recovery [30].

### 2.4. Polysomnography

All patients underwent level 1 or level 2 PSG preoperatively and at least 3 months postoperatively. PSG recordings were manually checked and scored according to the standards of the American Academy of Sleep Medicine (AASM) Manual for the Scoring of Sleep and Associated Events [4]. The collected preoperative and postoperative PSG variables included AHI, AI, HI, oxygen desaturation index (ODI), and lowest oxyhemoglobin saturation (LSAT).

Patients were designated into the apnea-predominant OSA group (AP-group) whenever the AHI ≥5 events/hour and more than 50% of the AHI consisted of apneas (AI/AHI >50%). Patients presenting with an AHI of ≥5 events/hour and with apneas less than 50% of the AHI (AI/AHI <50%), were allocated to the hypopnea-predominant OSA group (HP-group) [31,32,33]. Patients with the ratio of AHI during rapid eye movement (REM) sleep (REM-AHI) to AHI during non-REM sleep (NREM-AHI) >2 and NREM-AHI <15 events/hour were classified as REM-related OSA [34]. Positional OSA was defined as an AHI at least twice as high in supine position as in non-supine position [35].

Surgical success was defined according to Sher’s criteria, with an AHI reduction of at least 50% and an AHI below 20 events/hour postoperatively [36]. Patients meeting the criteria for surgical success were referred to as responders. Surgical cure was defined as a postoperative AHI below 5 events/hour [37].

### 2.5. Cephalography

**Figure 1:**
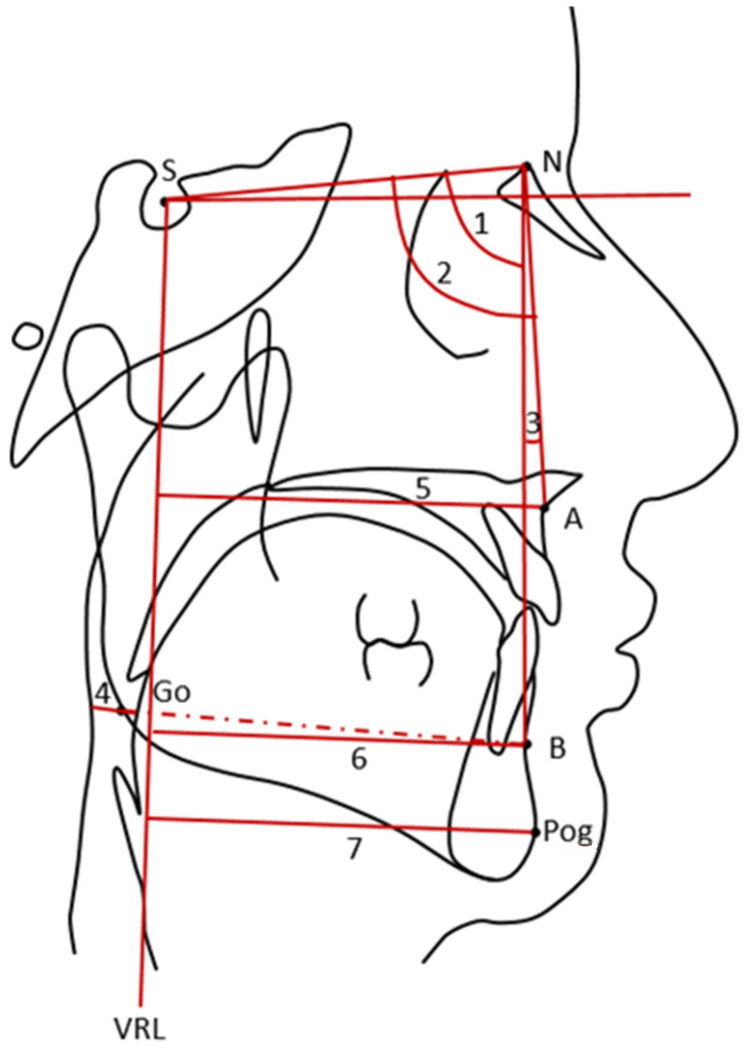
Cephalometric measurements. Landmark and reference line: A, A-point; B, B-point; Go, gonion; N, nasion; Pog, pogonion; S, sella; VRL, vertical reference line. Variables: 1, SNA, angle from sella to nasion to A-point (degree); 2, SNB, angle from sella to nasion to B-point (degree); 3, ANB, angle from A-point to nasion to B-point (degree); 4, PAS, posterior airway space (mm); 5, A-point to vertical reference line (mm); 6, B-point to vertical reference line (mm); 7, pogonion to vertical reference line (mm).

A standard lateral cephalogram was obtained on all patients before and at least one week after MMA. All cephalograms were traced by one examiner using the Viewbox (version 4; dHAL Software, Kifissia, Greece). The following baseline cephalometric measurements were obtained: SNA (angle from sella to nasion to A-point), SNB (angle from sella to nasion to B-point), ANB (angle form A-point to nasion to B-point), and posterior airway space (PAS; distance between the base of the tongue and the posterior pharyngeal wall, derived from a line connecting B-point to gonion). Degree of maxillary advancement was traced from A-point displacement with respect to the vertical reference line (VRL). Degree of mandibular advancement was traced from B-point and pogonion (pog) with respect to VRL, respectively (Figure 1).

### 2.6. Excessive Daytime Sleepiness

Excessive daytime sleepiness as one of the main symptoms and burden of OSA, was scored preoperatively with the use of the Epworth Sleepiness Scale questionnaire (ESS) [38]. This self-administered questionnaire contains 8 questions, to which respondents were asked to rate on a 4-point Likert-scale (0–3).

### 2.7. Statistical Analysis

Statistical analysis was performed using SPSS (version 26.0; IBM Corp., Armonk, NY, USA). Continuous variables were reported as the mean and standard deviation (SD) when data were normally distributed or median and interquartile range (IQR) when data were not normally distributed. Categorical variables were reported as frequency and percentage. Normality was tested using the Shapiro–Wilk test. To compare baseline characteristics between AP-group and HP-group, the independent *t*-test was used in case of normally distributed data and the Mann–Whitney U test was used in case of non-normally distributed data. To compare the paired continuous data, the paired-sample t-test was used when data were normally distributed, while the Wilcoxon signed rank test was used when data were not normally distributed. The chi-squared test was used to compare the rates of surgical success and cure between groups. To correct for possible confounders in surgical outcome, a multivariate logistic regression analysis was performed. Linear regression analysis was performed to investigate the association between pre-op ESS and apnea and/or hypopnea-predominant OSA group. For all analyses, a *p* value < 0.05 was considered statistically significant.

## 3. Results

At the department of Oral and Maxillofacial Surgery at the Amsterdam UMC, location AMC, a total of 114 patients underwent MMA for OSA, between 2010 and 2021. Of those patients eighteen were excluded due to the following reasons: No consent from the patient for the use of their data for research purposes (*n* = 3), incomplete pre- and/or postoperative PSG data (*n* = 9), mild OSA (*n* = 4), and other adjunctive procedures performed at the time of MMA (*n* = 2). Therefore, 96 patients were included in this study, 77 males (80.2%) and 19 females (19.8%). The mean age was 50.9 ± 9.9 years. The median body mass index (BMI) was 29.7 (27.4–32.2) kg/m^2^. A detailed overview of baseline characteristics of the total population is presented in Table 1.

### 3.1. Baseline Characteristics of AP-Group Versus HP-Group

When comparing baseline characteristics between AP-group and HP-group, the percentage of patients who received previous upper airway surgery was significantly higher in AP-group compared to HP-group (*p* = 0.034). The baseline AHI (*p* < 0.001), AI (*p* < 0.001), and ODI (*p* < 0.001) were significantly higher in AP-group, while HI was significantly higher in HP-group (*p* < 0.001). Additionally, the percentage of positional OSA was significantly higher in HP-group compared to AP-group (*p* = 0.012). There was no significant difference between groups in the other baseline characteristics (Table 1).

### 3.2. MMA Surgical Outcome

In the total population, the mean degree of A-point advancement, mean degree of B-point advancement, and median degree of pog advancement were 7.3 ± 2.3 mm, 10.0 ± 4.3 mm, and 9.2 [6.6, 12.5] mm, respectively. No significant difference was found between AP-group and HP-group in the advancement degrees of A-point, B-point, and pog (Table 2).

For the AP-group, except for median HI, the median AHI (*p* < 0.001), median AI (*p* < 0.001), median ODI (*p* < 0.001), and median LSAT (*p* < 0.001) were all significantly improved after MMA. For the HP-group, the median AHI (*p* < 0.001), median AI (*p* < 0.001), median HI (*p* < 0.001), median ODI (*p* < 0.001), and median LSAT (*p* = 0.005) were all significantly improved after MMA. An overview of preoperative and postoperative PSG variables of both groups are shown in Table 3.

Surgical success was achieved in 39 of 68 patient (57.4%) in the AP-group and 23 of 28 patients (82.1%) in the HP-group. Surgical cure was achieved in 9 patients (13.2%) in the AP-group and 10 patients (55.5%) in the HP-group. Both surgical success rate (*p* = 0.021) and cure rate (*p* = 0.012) were significantly higher in the HP-group. Multivariate logistic regression analysis showed no significant association between surgical success and gender (odds ratio [OR] = 1.748, 95% confidence interval [CI] = 0.486–6.287; *p* = 0.393), age (OR = 0.957, 95% CI = 0.911–1.004; *p* = 0.073), baseline BMI (OR = 1.030, 95%CI = 0.921–1.151; *p* = 0.608), previous upper airway surgery (OR = 0.735, 95%CI = 0.293–1.845; *p* = 0.512), and baseline AHI (OR = 0.999, 95% CI = 0.978–1.021; *p* = 0.957).

### 3.3. Postoperative Shift in Apnea/Hypopnea Predominance

Of the total population, 68 patients (70.8%) were apnea-predominant preoperatively, 37 of the 68 patients (54.4%) shifted to hypopnea- predominant after MMA. Twenty-eight patients (29.2%) were hypopnea-predominant preoperatively, 7 of them (25%) shifted to apnea-predominant postoperatively. After MMA, the percentages of apnea-predominant and hypopnea-predominant were 39.6% (*n* = 38) and 60.4% (*n* = 58), respectively.

### 3.4. Responders Versus Non-Responders

**Figure 2:**
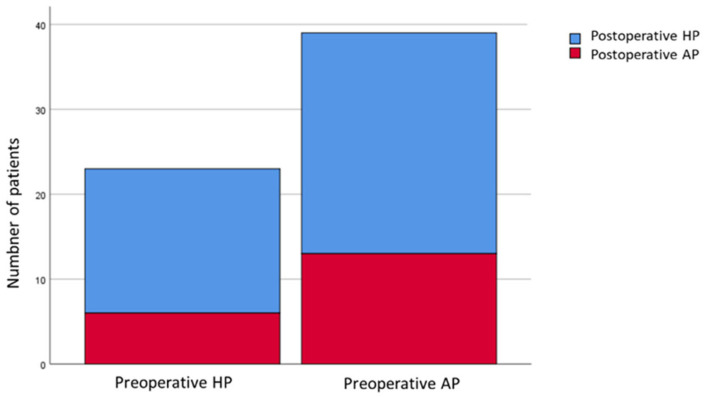
Distribution of apnea/hypopnea predominance in responders. AP, apnea-predominant; HP, hypopnea-predominant.

In the responder group (*n* = 62), 39 patients (62.9%) were apnea-predominant preoperatively. Twenty-six of preoperative apnea-predominance (66.7%) changed to postoperative hypopnea-predominance. Among the 23 hypopnea-predominance preoperatively (37.1%), 6 (26.1%) changed to apnea-predominance postoperatively (Figure 2).

**Figure 3:**
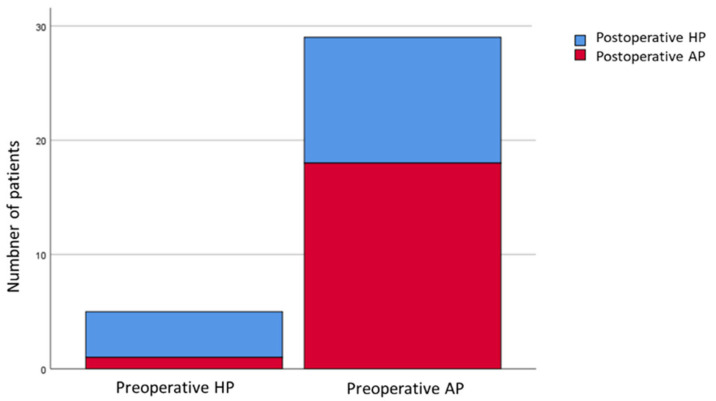
Distribution of apnea/hypopnea predominance in non-responders. AP, apnea-predominant; HP, hypopnea-predominant.

In the non-responder group (*n* = 34), 29 patients (85.3%) were apnea-predominant preoperatively. Eleven of preoperative apnea-predominance (32.4%) shifted to hypopnea-predominance postoperatively. Only 5 patients (14.7%) were hypopnea-predominant preoperatively, 1 patient (20%) changed to apnea-predominance after MMA (Figure 3).

### 3.5. Excessive Daytime Sleepiness in Apnea-Predominant vs. Hypopnea-Predominant OSA

The ESS was preoperatively scored by a minority of participants (*n* = 43) (Table 1). The mean preoperative ESS for the AP-group (*n* = 32) and HP-group (*n* = 11), was 13.0 ± 6.1 and 9.8 ± 5.8, respectively. When adjusting for potential confounders—such as age, gender, BMI, AHI, ODI, and LSAT—linear regression analysis showed that AP-group was significantly associated with a higher ESS (*p* = 0.008).

## 4. Discussion

This study set out with the aim of assessing whether hypopnea-predominant OSA was more likely to achieve surgical success and to have better resolution of PSG parameters when compared to apnea-predominant OSA after MMA. The second aim in this study was to investigate whether MMA alters the presence of apnea-predominant OSA to hypopnea-predominant OSA or vice versa. As far as the authors are aware this is the first study that has specifically looked into these issues related to MMA outcome.

The most obvious finding to emerge from the analysis was that although MMA was able to significantly reduce the AHI in both the AP-group as well as the HP-group, MMA achieved a significantly better surgical success and cure rate in the HP-group compared to the AP-group. Additionally, MMA was able to significantly improve the median AHI, median AI, median HI, median ODI, and median LSAT in the HP-group. This might suggest that patients with hypopnea-predominant OSA might be more suitable candidates for MMA compared to patients presenting with apnea-predominant OSA. However, with a small sample size (28 patients in HP-group), caution must be applied here. Moreover, Mattew et al. found that a majority of extremely obese patients manifest with a preponderance of hypopneas [39]. When looking at the results of this study, this would propose that extremely obese patients would be more suitable candidates for MMA, which is in stark contrast to previous research, which established that a lower BMI was associated with a higher MMA success rate [19].

It was interesting to see that the number of patients who received previous upper airway surgery was significantly higher in the AP-group. Holty et al. reported in their review that they found that patient who underwent previous phase -1 surgery—uvulopalatopharyngoplasty—were less likely to achieve surgical cure after MMA [19]. The presence of apnea-predominant OSA in patients who received previous upper airway surgery, might explain why MMA is less successful in these patients. However, more research is necessary and needs to be carried out in order to confirm and possibly also explain this finding. It is important to bear in mind that many factors of course, may lead to MMA failure in patient who underwent previous phase -1 surgery. In contrast, Tang et al. looked into treatment outcome in apnea and hypopnea-predominant OSA patients undergoing adenotonsillectomy, and they found that there was no difference in outcome whether patients had apnea-predominant or hypopnea-predominant OSA [40]. This may be explained by the fact that non-anatomical factors (e.g., neuromuscular activation, ventilatory control, and arousal threshold) in children contribute significantly, and therefore anatomical airway factors might play a small role in the contribution to ventilatory instability and obstructive cycling of OSA [41].

Another important finding was that a majority of patients tended to shift from the AP-group prior to MMA to the HP-group after MMA. A possible explanation for this might be that MMA converted some apneas to hypopneas in these patients. There were 7 patients, which shifted from the HP-group prior to MMA to the AP-group after MMA. In all 7 patients, the AHI was significantly reduced postoperatively. While in these 7 patients MMA showed to be more effective in the reduction in the hypopneas than the apneas, 3 patients showed an increase in apneas after MMA. An analysis of non-anatomical factors (e.g., Pcrit, loop gain, muscle responsiveness, and arousal threshold) might shed more light on these different outcomes, the mechanisms responsible for the different types of apneas and hypopneas, and it might explain the role of the apnea and hypopnea component in those patients, which were not successfully treated with MMA. A study on a non-framework surgery in patients with very severe OSA showed similar findings with our study. It was found that in addition to frequency, duration of hypopnea could also increase in patients—with AHI ≥ 60 events/h—after one-stage multi-level sleep surgery [42]. These results emphasize the fact that MMA often not only improves the patient’s severity of OSA, but also alters the patients type of OSA. Previous studies also highlighted this effect, by illustrating that MMA was able to alter the presence of positional dependency and percentage of central and mixed apnea index [23,24]. This observation suggests that it is likely that the mechanisms responsible for the different types of apneas overlap.

When looking at definition of the American Academy of Sleep Medicine for a apnea —decline of the peak signal excursion by ≥90% of pre-event baseline for ≥10 s—and hypopnea—decline of the peak signal excursions by ≥30% of pre-event baseline for ≥10 s in association with either ≥3% arterial oxygen desaturation or an arousal—one might hypothesize that patients presenting with apnea-predominant OSA might experience more OSA related burden compared to patients with hypopnea-predominant OSA [4,5,43]. In order to investigate this, we evaluated the ESS of patient prior to MMA, and divided them into an apnea-predominant and hypopnea-predominant OSA group. The results illustrate that apnea-predominant OSA is significantly associated with more excessive daytime sleepiness. When looking at the non-responders, it was found that 32% of patients shifted from apnea-predominant OSA to hypopnea-predominant OSA, the case can be made that even though these patients did not respond to MMA as one might have hoped for, MMA might still have alleviated some of these patients OSA burden by shifting them from apnea-predominant OSA to hypopnea-predominant OSA.

A note of caution is due here when interpreting the results of this study since there are several limitations. For one, the present study is a single-center retrospective cohort study. There are 12 patients who were excluded from the analysis due to the patient’s rejection for the use of their data for research purposes or incomplete pre- and/or postoperative PSG data; therefore, there is an inherent concern for potential selection bias. Secondly, the study population could be regarded as small. With this small sample size, caution must be applied, due to the fact that this could potentially lead to sampling bias [44]. Thirdly, only 45% of the study participants completed the ESS, which therefore introduces the possibility for non-response bias [45]. Finally, there is some controversy surrounding hypopneas. This is partially based on the fact that there have been many different definitions for hypopnea over time [46]. Additionally, it is believed that scoring of hypopneas can be difficult and therefore lead to disagreements in scoring of apneas vs. hypopneas [47]. Previous papers have also mentioned the fact that specifically for hypopneas it is difficult to distinguish between obstructive and central events [39]. These controversies and difficulties could have influenced the data collected for this study and therefore influenced our findings. Further research should be undertaken in order to confirm the present findings. The authors advocate for larger prospective multicenter studies, where specific attention is paid to the scoring for both apneas and hypopneas.

In addition to MMA, there are other evidence-based treatment options for OSA. Mandibular advancement device (MAD) is the most common oral appliance that offers a non-invasive option for the management of OSA. It needs to be noted that, in nature, MAD can only alleviate OSA-related symptoms, while MMA can treat OSA. Therefore, MMA can provide a solution for OSA patients who decline to accept lifelong treatment with MAD. Moreover, in order to further assist decision making in OSA treatment, further research on cost-effectiveness of MMA in OSA treatment is necessary.

In spite of its limitations, the authors strongly feel that the study adds to our understanding that specific characteristics of preoperative PSG—other than the AHI and in this case specifically apnea or hypopnea predominance—might be useful in predicting MMA outcome. This can aid physicians to better select candidates for MMA in order to increase surgical treatment successes, and better utilize the growing scarcity of medical resources.

## 5. Conclusions

Notwithstanding the study limitations, the findings suggest that patients presenting with hypopnea-predominant OSA might be associated with better surgical response after MMA. In addition, our results further support the concept that MMA is able to alter patients OSA phenotype.

## Figures and Tables

**Table 1:** Baseline characteristics of the total population, AP-group and HP-group.

	Total Population (*n* = 96)	Preoperative AP-Group (*n* = 68)	Preoperative HP-Group (*n* = 28)	*p*-Value
Age (years)	50.9 ± 9.9	50 [44, 58]	53.5 [46.3, 59.8]	0.631
Gender (*n*, %)				
Female	19 (19.8%)	11 (16.2%)	8 (28.6%)	0.166
Male	77 (80.2%)	57 (83.8%)	20 (71.4%)	
BMI (kg/m^2^)	29.7 [27.4, 32.2]	29.6 [27.2, 31.6]	30.3 [27.9, 33.3]	0.124
Previous upper airway surgery (*n*, %)				
Yes	40 (41.7%)	33 (48.5%)	7 (25%)	0.034
No	56 (58.3%)	35 (51.5%)	21 (75%)	
SNA (degree)	80.0 ± 3.9	79.9 ± 4.2	80.4 ± 3.0	0.575
SNB (degree)	75.2 [72.2, 78.4]	75.3 [72.2, 78.1]	75.2 [73.4, 79.4]	0.424
ANB (degree)	4.9 [2.8, 6.7]	5.1 [2.9, 6.7]	4.4 [2.7, 6.1]	0.452
PAS (mm)	9.0 ± 3.1	9.1 ± 3.1	8.7 ± 3.0	0.163
AHI (events/hour)	52.7 (21.1)	58.7 [41.9, 73.6]	36.4 [26.3, 49.8]	<0.001
AI (events/hour)	34.1 [17.1, 60.2]	48.2 ± 20.5	12.1 ± 8.3	<0.001
HI (events/hour)	12.5 [4.0, 22.9]	7.6 [3.1, 16.4]	23.7 [19.1, 30.8]	<0.001
Positional OSA (*n*, %)				
Yes	31 (38.3%)	20 (31.1%)	11 (64.7%)	0.012
No	50 (61.7%)	44 (68.8%)	6 (35.3%)	
REM-related OSA (*n*, %)				
Yes	1 (1.2%)	0 (0)	1 (5.6%)	0.220
No	81 (98.8%)	64 (100%)	17 (94.4%)	
ODI (events/hour)	52.1 ± 21.3	56.5 ± 21.0	37.1 ± 15.1	<0.001
LSAT (%)	80 [73.0, 84.0]	79 [71.5, 84]	82 [78.0, 87.0]	0.165
ESS (score)	12.2 ± 6.1	13.0 ± 6.1	9.8 ± 5.8	0.138

Continuous data are presented as mean ± standard deviation or median [Q1, Q3]. Categorical data are presented as frequency and percentage. *P* values comparing AP and HP; *p* < 0.05 is considered statistically significant. AHI, apnea hypopnea index; AI, apnea index; ANB, angel form A-point to nasion to B-point; AP-group, apnea-predominant OSA group; BMI, body mass index; ESS, Epworth Sleepiness Scale; HI, hypopnea index; HP-group, hypopnea-predominant OSA group; OSA, obstructive sleep apnea; LSAT, lowest oxyhemoglobin saturation; ODI, oxygen desaturation index; PAS, posterior airway space; REM, rapid eye movement; SNA, angel from sella to nasion to A-point; SNB, angel from sella to nasion to B-point.

**Table 2:** Surgical characteristics of the total population, AP-group and HP-group.

	Total Population (*n* = 96)	AP-Group (*n* = 68)	HP-Group (*n* = 28)	*p*-Value
A-point advancement (mm)	7.3 ± 2.3	7.2 ± 2.0	7.4 ± 2.9	0.746
B-point advancement (mm)	10.0 ± 4.3	9.8 ± 4.1	10.4 ± 4.9	0.548
Pog advancement (mm)	9.2 [6.6, 12.5]	9.1 [5.9, 12.2]	10.0 [7.5, 13.0]	0.408

Data are presented as mean ± standard deviation or median [Q1, Q3]. *P* values comparing AP-group and HP-group; *p* < 0.05 is considered statistically significant. AP, apnea-predominant OSA group; HP, hypopnea-predominant OSA group; mm, millimeters.

**Table 3:** Preoperative and postoperative polysomnography values in AP-group and HP-group.

AP-Group (*n* = 68)			
	Preoperative	Postoperative	*p*-Value
AHI (events/hour)	58.7 [41.9, 73.6]	13.7 [7.3, 24.2]	<0.001
AI (events/hour)	46 [30.1, 64.4]	6.1 [1.9, 14.3]	<0.001
HI (events/hour)	7.6 [3.1, 16.4]	6.4 [3.8, 10.6]	0.308
ODI (events/hour)	56.9 [40.3, 74.9]	21.8 [10.9, 32.5]	<0.001
LSAT (%)	79 [71.5, 84]	85.5 [82, 89]	<0.001
**HP-Group (*n* = 28)**			
	**Preoperative**	**Postoperative**	***p*-Value**
AHI (events/hour)	36.4 [26.3, 49.8]	9.9 [4.5, 18.9]	<0.001
AI (events/hour)	10.7 [5.3, 17.2]	1.5 [0.9, 6.8]	<0.001
HI (events/hour)	23.7 [19.1, 30.8]	5.3 [3.0, 15.5]	<0.001
ODI (events/hour)	37.1 ± 15.1	17.8 ± 11.7	<0.001
LSAT (%)	82 [78, 87]	87 [82, 88.8]	0.005

Data are presented as mean ± standard deviation or median [Q1, Q3]. *P* values comparing preoperative and postoperative values; *p* < 0.05 is considered statistically significant. AHI, apnea hypopnea index; AI, apnea index; AP-group, apnea-predominant OSA group; HI, hypopnea index; HP-group, hypopnea-predominant OSA group; LSAT, lowest oxyhemoglobin saturation; ODI, oxygen desaturation index.

## Data Availability

The data presented in this study are available on request from the corresponding author.
